# Supplementary material for: Sleeve Gastrectomy Improves Hepatic Glucose Metabolism by Downregulating FBXO2 and Activating the PI3K-AKT Pathway
Source: Int J Mol Sci. 2023 Mar 14;24(6):5544. doi: 10.3390/ijms24065544 (PMC10052132; doi:10.3390/ijms24065544)
Supplement: Supplementary file 1 [file ijms-24-05544-s001.zip › ijms-2235705-supplementary.pdf]

International Journal of *Molecular Sciences*

MDPI

Supplementary Table S1. The antibodies for Western blot.

Name	Company	Product Number	Source	MW (kDa)	Dilution
GAPDH	proteintech	60004-1-lg	Mouse	36	1:5000
AMPKa	Cell Signaling	#2793	Mouse	62	1:1000
p-AMPKa	Cell Signaling	#2535	Rabbit	62	1:1000
mTOR	Cell Signaling	#2983	Rabbit	286	1:1000
p-mTOR	Cell Signaling	#5536	Rabbit	286	1:1000
IR	Cell Signaling	#3025	Rabbit	95	1:1000
PI3K	Cell Signaling	#4249	Rabbit	110	1:1000
ATK	Cell Signaling	#4685	Rabbit	60	1:1000
P-AKT	Cell Signaling	#4060	Rabbit	60	1:1000
GSK3β	proteintech	#12456	Rabbit	46	1:1000
p-GSK3β	Cell Signaling	#5558	Rabbit	46	1:1000
FBXO2	proteintech	14590-1-AP	Rabbit	40	1:1000

glyceraldehyde-GAPDH, glyceraldehyde-3-phosphate dehydrogenase; AMPa, AMP-activated protein kinase alpha subunit; m-TOR, mechanistic target of rapamycin kinase; IR, insulin receptor; PI3K, phosphatidylinositol 3-kinase; AKT, Akt kinase; GSK3β, glycogen synthase kinase 3 beta; FBXO2, F-box protein 2. "p": Phospho-.

Supplementary Table S2. The primer sequences for Q-PCR.

Gene name	Species	sequences	
GAPDH	Human	Forward Primer	GGAGCGAGATCCCTCCAAAAT
		Reverse Primer	GGCTGTTGTCATACTTCTCATGG
FBXO2	Human	Forward Primer	GTGTCGCAAAGCACAGGTC
		Reverse Primer	CGGACAGTAGCTTAACGGTGAG
Gapdh	Mouse	Forward Primer	ACAAGCTCACGTACTCCACTG
		Reverse Primer	TCCTCCATAGTAAGGCCACATC
Fbxo2	Mouse	Forward Primer	GGTTCCGCTCAATGGTATCAA
		Reverse Primer	TGCCCTGGTACTTCCTATCTTC
Fasn	Mouse	Forward Primer	GGAGGTGGTGATAGCCGGTAT
		Reverse Primer	TGGGTAATCCATAGAGCCCAG
Srebp1	Mouse	Forward Primer	TGACCCGGCTATTCCGTGA
		Reverse Primer	CTGGGCTGAGCAATACAGTTC
Acly	Mouse	Forward Primer	ACCCTTTCACTGGGGATCACA
		Reverse Primer	GACAGGGATCAGGATTTCCTTG
Dgat1	Mouse	Forward Primer	TCCGTCCAGGGTGGTAGTG
		Reverse Primer	TGAACAAAGAATCTTGCAGACGA
Pparg	Mouse	Forward Primer	TCGCTGATGCACTGCCTATG
		Reverse Primer	GAGAGGTCCACAGAGCTGATT
Apoa1	Mouse	Forward Primer	GGCACGTATGGCAGCAAGAT
		Reverse Primer	CCAAGGAGGAGGATTCAAACTG
Apoae	Mouse	Forward Primer	CTGACAGGATGCCTAGCCG
		Reverse Primer	CGCAGGTAATCCCAGAAGC
Apoab	Mouse	Forward Primer	AAGCACCTCCGAAAGTACGTG
		Reverse Primer	CT CCAGCT CTACCTTAuAGTT GA
Cd36	Mouse	Forward Primer	ATGGGCTGTGATCGGAACTG
		Reverse Primer	GTCTTCCCAATAAGCATGTCTCC
Fabp1	Mouse	Forward Primer	ATGAACTTCTCCGGCAAGTACC
		Reverse Primer	CTGACACCCCCTTGATGTCC
Foxo1	Mouse	Forward Primer	CCCAGGCCGGAGTTTAACC
		Reverse Primer	GTTGCTCATAAAGTCGGTGCT
Irs1	Mouse	Forward Primer	CGATGGCTTCTCAGACGTG
		Reverse Primer	CAGCCCGCTTGTTGATGTTG

Irs2	Mouse	Forward Primer	CTGCGTCCTCTCCCAAAGTG
		Reverse Primer	GGGGTCATGGGCATGTAGC
P6gc	Mouse	Forward Primer	CGACTCGCTATCTCCAAGTGA
		Reverse Primer	GTTGAACCAGTCTCCGACCA
Pck1	Mouse	Forward Primer	CTGCATAACGGTCTGGACTTC
		Forward Primer	CAGCAACTGCCCGTACTCC
Fbp1	Mouse	Forward Primer	TATGGTGGAAAGGGACGGGAA
		Reverse Primer	CCTCTGGTGATACTCAAGGATGG

GAPDH, glyceraldehyde-3-phosphate dehydrogenase; FBXO2, F-box protein 2; Fasn, fatty acid synthase; Dgat1, diacylglycerol O-acyltransferase 1; Acly, ATP citrate lyase; Srebp1, sterol regulatory element binding protein 1; Pparg, Peroxisome proliferator-activated receptor gamma; Apoa1, apolipoprotein A1;Apob, apolipoprotein B; Apoe, apolipoprotein E; Cd36, CD36 molecule; Fabp1, fatty acid binding protein 1; Foxo1, forkhead box O1; Irs, insulin receptor substrate; G6pc, glucose-6-phosphatase, catalytic; Pck1, phosphoenolpyruvate carboxykinase 1; Fbp1, fructose-bisphosphatase 1.

Supplementary Figure S1

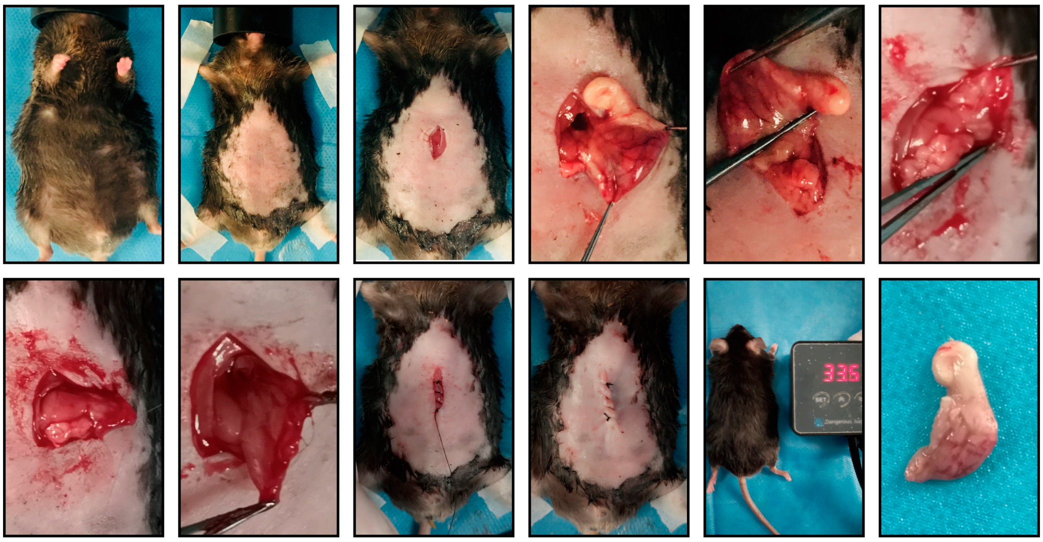

Supplementary Figure S1. Details of bariatric surgery operations.

Supplementary Figure S2

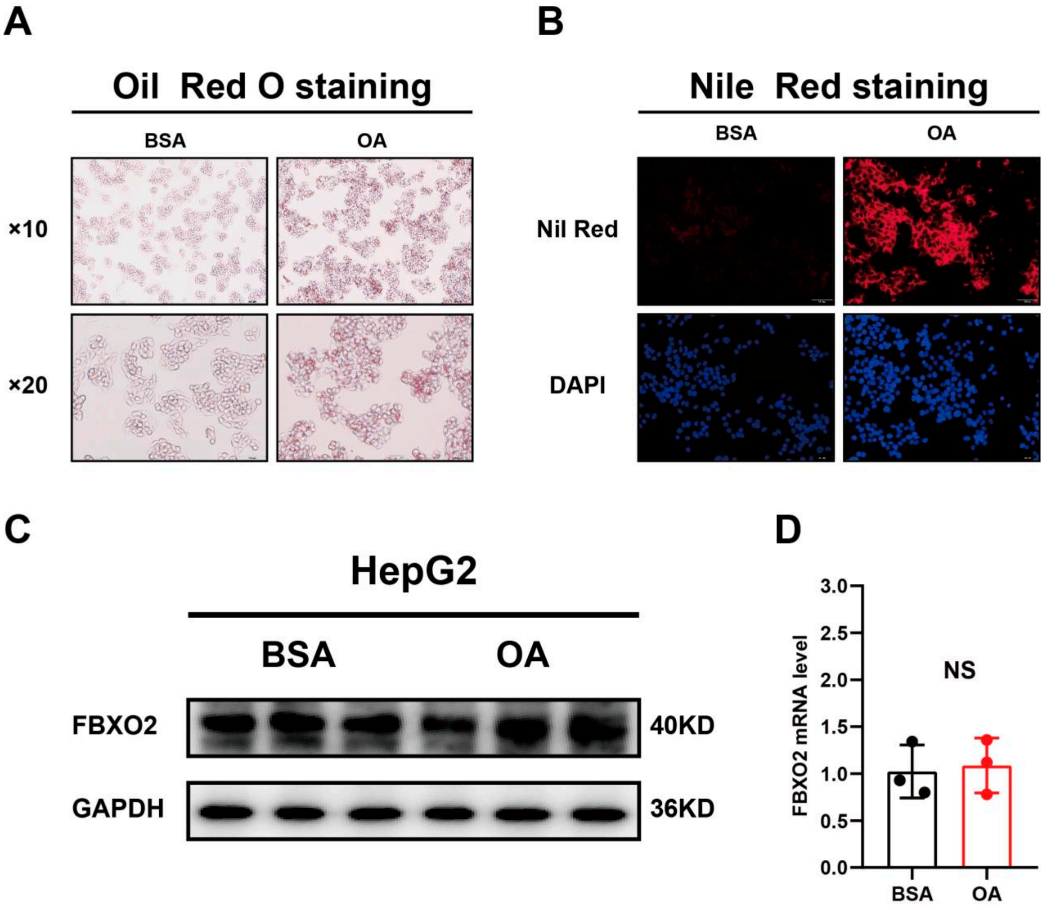

Supplementary Figure S2. FBXO2 expression was not affected by circulating fatty acid concentration. (A) Oil Red O staining and (B) Nil Red staining of HepG2 cells treated with odium oleate.(Scale bar, 10×, 200μm; 20×, 100 μm). (C) Western blot result and PCR result (D) of protein and relative mRNA levels of FBXO2 in HepG2 cells in each group (n=3).
